# Parenting practices that may encourage and discourage physical activity in preschool-age children of Brazilian immigrant families: A qualitative study

**DOI:** 10.1371/journal.pone.0214143

**Published:** 2019-03-20

**Authors:** Ana Cristina Lindsay, Carlos André Moura Arruda, Gabriela Perreira De Andrade, Márcia Maria Tavares Machado, Mary L. Greaney

**Affiliations:** 1 Department of Exercise and Health Sciences, University of Massachusetts, Boston, MA, United States of America; 2 Department of Community Health–School of Medicine, Federal University of Ceará, Fortaleza, Brazil; 3 Health Studies & Department of Kinesiology, University of Rhode Island, Kingston, RI, United States America; University of Bristol, UNITED KINGDOM

## Abstract

Brazilians are a rapidly increasing Latino immigrant group in the United States (US), yet little research has examined factors influencing physical activity (PA) levels and behaviors of children growing up in Brazilian immigrant families. This information is needed to develop culturally sensitive interventions tailored to this population. Therefore, this qualitative study explored PA parenting practices used by Brazilian immigrant mothers living in the US. Thirty-seven Brazilian immigrant mothers with at least one child between the ages of 2 and 5 years participated in 1of 7 focus group discussions. Thematic analysis identified seven parenting practices that mothers employ that may encourage or facilitate physical activity their preschool-aged children’s PA including: 1) modeling PA; 2) engaging and being physically active with child; 3) providing logistic support; 4) encouraging, praising, and offering motivational support; 5) watching, supervising, and teaching children how to engage in PA; 6) monitoring and setting limits to child’s screen time; and 7) prompting child to be physically active. In addition, analysis identified four parenting practices that may discourage or inhibit children’s PA including: 1) modeling of sedentary behaviors; 2) having rules and restrictions due to safety- and weather-related concerns; 3) limiting child’s outdoor time due to parental time constraints; and 4) restricting child’s outdoor and play time as punishment. Furthermore, analyses demonstrated that social contextual factors (e.g., income, housing, neighborhood safety, etc.) influence mothers’ PA parenting practices and consequently, their children’s PA. This is the first qualitative study, to our knowledge, to explore PA parenting practices of Brazilian-born immigrant mothers living in the US. Future research should further explore PA parenting practices of Brazilian immigrant parents including quantifying PA parenting practices that encourage and discourage PA, as well as examining the influence of fathers’ PA parenting practices on young children’s PA.

## Introduction

Physical activity (PA) has important physical, emotional and social benefits for children [1–3]. Helping children develop the foundation for healthy PA habits is critical for health promotion and disease prevention in childhood and beyond [4–7]. Physically active children have healthier cardiovascular profiles, leaner body frames, and higher peak bone mass than physically inactive children [4,5]. In addition to regulating body weight and improving body composition, PA improves children’s psychological and social well-being [4,5].

Despite the well-documented benefits of PA, preschool-age (3–5 years) children’s PA levels have declined over the past decades, with approximately 50% of children in this age group in the United States (US) not getting the recommended 180 min (3 hours) of PA daily [8,9]. Furthermore, racial/ethnic minority children are less likely to meet the daily PA recommendations than white children [9,10]. Additionally, physical inactivity is even a greater problem among racial/ethnic minority children in the US [10–13], and Latino immigrant children have the highest prevalence of physical inactivity [14].

Parents play a unique and central role in influencing their children’s behaviors, including their PA habits [1–3], and this influence is particularly important during the habit-forming years of early childhood [1,15,16]. Existing scientific evidence suggests that one way in which parents influence their children’s PA levels and behaviors is through their parenting practices [2,3,17]. Parenting practices are the specific sets of behaviors that characterize parents’ interactions with their children, and the beliefs and attitudes that underpin these interactions [18–21]. PA parenting practices describe what parents do to encourage or facilitate their children’s PA, such as providing logistic support for PA, encouraging their children to be physically active (e.g., verbal encouragement), monitoring and setting limits for sedentary behaviors (e.g., limiting screen time), modeling PA behaviors, etc. [2,20–24]. Moreover, evidence indicates that some parenting practices such as having rules and restrictions (e.g., no running inside, no playing outside due to safety concerns, etc.), psychological control (e.g., parental criticism and insults), etc. may unintentionally discourage or inhibit children’s PA [3,22,23].

The social contextual model [25], an adaptation of the socio-ecological model (SEM), integrates social class and culture to the different levels of the SEM [26], including (a) intrapersonal or individual factors (material circumstances, daily hassles); (b) interpersonal factors (social support, networks); (c) organizational factors (healthcare services, and programs, health insurance); and (d) environmental factors (access and proximity to parks and playgrounds). The social contextual model emphasizes the expression of cultural pathways that may influence/impact behaviors and explicitly inform the design of health promotion interventions [25]. The social context, cultural norms and values influence parental beliefs, attitudes, and practices toward child rearing thus parenting practices may vary across ethnic groups [27,28]. Research is needed to understand how culture influences parenting practices so interventions can best address these practices.

Brazilians are a rapidly increasing Latino immigrant group in the US, yet little research has examined factors influencing PA levels and behaviors of Brazilian children growing up in immigrant families [29–31]. This information is needed to develop culturally sensitive interventions tailored to this population. Therefore, the purpose of this qualitative study was to explore PA parenting practices used by Brazilian immigrant mothers of preschool-aged children.

## Methods

This study was conducted in two cities located in Massachusetts (MA): Somerville and Everett. The present qualitative study was part of a larger ongoing mixed-methods research study (to date 137 unique families) with Brazilian families living in the Greater Boston area examining parenting styles and parenting practices (e.g., promoting healthy eating, PA, adequate sleep, and limiting screen time) related to the risk of childhood obesity [29,30,32,33].

Focus group discussions (FGDs) were used to gain an in-depth understanding of parenting practices of Brazilian-born immigrant mothers living in the US that may promote or prevent their preschool-aged children from being physically active. In addition, the guide explored mothers’ information seeking about PA and screen time, and beliefs and practices related to sleep and bedtime routines of their preschool-aged children. These results are presented elsewhere [29,30]. FGDs are valuable techniques for working in diverse cultural settings as they yield rich information [34] as the synergistic effects of the group settings elicit ideas and discussion that may not arise in individual interviews [34]. This study received ethical approval from the University of Massachusetts–Boston Ethics Board (IRB # 2013060).

### Participants

Participants were recruited through flyers posted at local Brazilian businesses and community-based social and health services agencies, as well as through announcements and events at two predominantly Brazilian churches. Interested participants called the phone number listed on the flyer or spoke to study staff at church events. Participants also were recruited using a snowball sampling technique [35], with women enrolled in the study asking their Brazilian friends with preschool-aged children if they would be interested in participating in the study.

Women were eligible to participate if they had at least one child aged 2–5 years, were of Brazilian ethnicity, born in Brazil, and had been living in the US for at least 12 months. We enrolled a convenience sample of 37 Brazilian immigrant mothers into the study between March and August 2017, seven of whom were recruited through the use of snowball sampling technique.

### Data collection

A native Brazilian-Portuguese speaker (ACL) trained in qualitative research methods moderated all FGDs in Portuguese using a semi-structured discussion guide that was developed based on previous systematic reviews of parenting practices [2,16,17] and explored participants’ views and experiences about: 1) how parents encourage or facilitate their preschool-age children to be physically active, and 2) how parents may unintentionally discourage or inhibit their preschool-age children from being physically active. In addition, the guide explored mothers’ information seeking about PA and screen time, and beliefs and practices related to sleep and bedtime routines of their preschool-aged children. These results are presented elsewhere [29,30]. The guide was piloted in a FGD with a small group of Brazilian immigrant mothers (n = 4) and then refined prior to use. Data from the pilot FGD were not included in the present study. Table 1 presents a sample of questions and prompts used in the FGDs.

**Table 1:** Sample of questions and prompts used in focus groups with mothers.

**Perceptions of and experience with child’s physical activity at home**
- Describe how important you think it is for children to be physically active.
○ *Probes*: *Do you think it is a problem for kids to spend too much time being* *sedentary or not being physically active*?
- Describe how your child is physically active at home.
○ *Probes*: *Plays actively outside*? *Plays actively inside*? *Does not play actively when at home*?
- Describe how satisfied you are with the amount of PA your child engages in while at home.
○ *Probes*: *Why*? *Do you wish he/she would be more active at home*? *Do you wish he/she had more outdoor playtime when at home*?
- Describe some types of PA or active play your child engages in while at home
○ *Probes*: *Riding his/her bike*? *Playing in nearby playground or park*?
- Describe some of your biggest concerns about how physically active your child is.
○ *Probes*: *Do you ever worry your child does not engage in enough PA*? *Do you ever worry that your child is too sedentary*?
- Describe what types of things would you like to change about how physically active your child is at home.
○ *Probes*: *Amount*? *Types of activity*?
**Parenting practices related to child’s physical activity**
- Do you think parents can influence how physically active their children are? How so?
- Describe what types of things you think parents can do to help their children be physically active.
- Describe what types things that you do to make sure or encourage your child to be physically active.
- Describe things that you do that you think might discourage your child to be physically active.
-Describe barriers that you face in making sure that your child is physically active when at home.
○ *Probes*: *Space*? *Time*? *Household obligations*? *Neighborhood safety*? *Knowledge of how physically active your child should be*? *Influence of other people in the household (e*.*g*. *older siblings*, *grandparents*, *father*, *mother)*?
-Describe barriers that you face in making sure that your child does not spend too much time sedentary time such as watching TV and/or videos or playing video games at home.
○ *Probes*: *Lack of household rules*? *Space*? *Time*? *Other obligations that keep you from having time to take your child out to play*?

Before each FGD, the moderator explained in Portuguese the study’s purpose, FGD procedures, study confidentiality, and obtained written informed consent from all participants. Before each FGD, participants were asked to think about their preschool-aged children when participating in the discussion. A trained, bilingual (Portuguese and English) research assistant (GDA) took notes during all FGDs, which were audio-recorded and lasted between 60–80 minutes. The moderator and research assistant met for about 15 minutes after each FGD to identify new and review recurring themes, which were entered into a grid that was used to closely follow emerging themes and to determine when data saturation was reached.

At the end of each FGD participants completed a brief, self-administered questionnaire in Portuguese that assessed education, marital status, access to health care services including participation in government-sponsored health and nutrition programs (e.g., WIC, Supplemental Nutrition Assistance Program (SNAP), etc.), country of origin, length of time living in the US, and acculturation, which was assessed via the Short Acculturation Scale for Hispanics (SASH), a 12-item measure scale validated for use in Latinos, including Mexican Americans, Cuban Americans, Puerto Ricans, Dominicans, and Central and South Americans. The SASH assesses language use, media use, and ethnic social relations [36], and items are measured on a scale of 1–5 (1 = least acculturated, 5 = fully acculturated), and an acculturation score was computed by averaging across the 12 items.

### Data analysis

The qualitative data was analyzed using thematic analysis, an iterative multistep process of coding data in phases to create meaningful patterns [37]. First, a professional transcriptionist and native Brazilian speaker transcribed all audio recordings verbatim in Portuguese. Second, two experienced qualitative researchers and native Portuguese speakers (ACL, CAMA) read several transcripts numerous times to become familiar with the content and generate initial codes [34,37]. Third, the two researchers manually coded all transcripts independently, but met regularly to discuss coding and to identify and resolve coding disagreements [37]. Fourth, coded text describing similar ideas were grouped and sorted to identify emergent themes and subthemes. Finally, salient text passages were extracted, and translated into English as illustrative quotes for emergent themes.

Although a thematic approach was used for the initial data analyses and the codebook did not include an a priori determined list of parenting approaches, it was evident during analysis that the self-described strategies were amenable to categorization based on well-recognized types of parenting practices [2,3,16,17,22,23,38]. Therefore, in a second phase of the data analyses, identified themes were sorted and grouped based on PA parenting practices identified in prior systematic reviews and studies [2,3,16,17,22,23,38]. In addition, descriptive statistics and frequencies were calculated for data collected in the socio-demographic survey using Microsoft Excel 2008.

## Results

Seven FGDs were conducted before saturation was reached, with no new themes or subthemes emerging during the final group. Mothers (n = 37) participating in this study were 26 to 41 (M = 35.3, SD = 2.8) years old. The majority was married (92%; n = 34), had graduated from high school (56.8%; n = 21), owned their own housecleaning business (92%; n = 34), and had two children (89%, n = 33). Approximately half (51%; n = 19) reported a family income of $40,000 or less, which is considered low-income for a family of four in the US, while the rest reported an annual income between 40,000 - $60,000, which is considered to be a low-middle income (Census Bureau). Participants were originally from three main regions of Brazil [e.g., the Southeast (e.g., Espirito Santo, Sao Paulo, and Minas Gerais), the South (e.g., Santa Catarina), the Midwest (e.g., Goias and Mato Grosso)], with the majority (59.5%; n = 22) being from the state of Minas Gerais, in the Southeast region. In addition, the majority spoke Portuguese at home (92%, n = 34), watched television programs in Portuguese (95%), and reported that the majority of their friends were Brazilians (87%). Mothers had lived in the US for an average of 6.7 (SD = 2.84) years, and their mean acculturation score was 1.43 (SD = 0.77), indicating that they identified more closely with Brazilian culture than with that of the US (S1 Table).

Identified themes were classified into three domains: 1) parenting practices that may encourage or facilitate children’s PA; 2) parenting practices that may discourage or inhibit children’s PA; and 3) social contextual factors affecting PA parenting practices. Although mothers with children aged 2–5 years were purposively recruited to participate in the study, most mothers discussed their PA parenting practices within the context of the entire family, including older children and fathers.

### Domain 1: Parenting practices that encourage or facilitate children’s physical activity

Analyses identified seven parenting practices, within this domain (see Table 2). These parenting practices included: 1) modeling PA; 2) engaging and being involved in PA with child; 3) providing logistic support; 4) encouraging, praising, and providing motivational support; 5) watching, supervising, and teaching children how to engage in PA; 6) monitoring and setting limits to child’s screen time; and 7) prompting child to be physically active.

**Table 2:** Parenting practices used by Brazilian immigrant mothers living in the United States that encourage and discourage their preschool-aged children to be physically active.

**Parenting Practices That Encourage of Facilitate Child’s Physical Activity (+)**	
**Themes**	**Subthemes**
Modeling physical activity	• Parental modeling of healthy PA behaviors
Engaging and being involved in physical activity with child	• Participating in PA with child • Being involved in child’s activities (e.g., coaching a sport)
Providing logistic support	• Providing transportation • Enrolling child in PA classes and sports • Taking child to park or playground, etc.
Encouraging, praising and providing motivational support	• Praising child for being physically active • Encouraging (e.g., you can do it, keep going, etc.) • Providing motivational support and positive reinforcement
Watching, supervising and teaching children how to engage in physical activity	• Watching child play outside • Supervising child’s outdoor time and active play • Teaching child how to engage in different types of activities
Monitoring and setting limits to child’s sedentary time	• Monitoring and setting limits on child’s screen time
Prompting child to be physically active	• Prompting child to be physically active by providing verbal or nonverbal encouragement for the child to engage in physical activity
**Parenting Practices That Discourage or Inhibit Child’s Physical Activity (—)**	
**Themes**	**Subthemes**
Modeling sedentary behaviors	• Parental modeling of sedentary behavior
Having rules and restrictions	• Having rules and restricting outdoor time and play due to safety concerns or weather-related reasons
Limiting children’s outdoor time and play due to parental time constraints	• Limiting child’s outdoor time and active play due to parental time constraints
Restricting child’s outdoor and play time as punishment or way of disciplining child	• Withholding outdoor time and active play as a means of disciplining child for bad behavior

#### Modeling physical activity

Across all FGD, all but one mother spoke of the importance of parents serving as PA role models and of this modeling having a positive influence on children’s PA. For example, mothers spoke of taking walks with their children, using exercising equipment at home, etc., and felt that these activities were important for exposing their children to PA. As one mother explained:

The most important thing a parent can do is to be a good model, and set a good example. We can’t expect children to be active if the parents are not active. So, I think the parents need to set a good example and be active themselves and the children will learn from them, will want to imitate them [parents]… FGD #2; Mother #12

#### Engaging and being involved in physical activity

Almost all mothers spoke of the importance of parents (mothers and fathers) being actively engaged and involved in their preschool-age children’s PA, and that this practice positively influenced children’s PA levels and behaviors. For example, mothers spoke of the importance of parents being active (e.g., going for walks or bike rides with children), playing sports such as soccer, baseball, etc. with their children, and that this fostered their children’s enjoyment of and desire to be physically active. One mother mentioned:

Parents who play with their children, play soccer, go for a walk, that’s important…to be active together… I think if a parent is involved and playing with their children, taking them to play soccer, ride a bike, that helps children being active… FGD #5; Mother #28

Furthermore, many mothers reported that their husbands or partners participated in more PA with their preschool-age children, especially with boys, than they did. This is illustrated on the following statements:

My husband coaches my son’s soccer team and I think it’s good because they both enjoy it [playing soccer] together… FGD #1; Mother #6

I have two boys and they love playing soccer. My husband also plays soccer and the boys love playing with him. During the weekend [Saturday] my husband has more time with the kids than I do, so they also spend a lot of time together … riding bikes, playing soccer, basketball… FGD #4; Mother #22

#### Providing logistic support

About two-thirds of mothers spoke of enrolling their preschool children in sports classes, taking children to the park, buying active toys (e.g., playground sets, small trampolines), providing transportation and that viewed these practices as important for promoting PA among their preschool-aged children. As one mother reported:

The YMCA near our house offers some classes [physical activity and sports] for young children &I enrolled my son [5 year old] in an all sports class and he loved it! He is now doing soccer… FGD #1; Mother #4

Moreover, some mothers spoke of their network of friends (e.g., other Brazilian parents) positively influencing their children’s PA by their provision of informational and/or logistic support. For example, mothers mentioned that having friends tell them about PA classes and organized sports classes, motivated them to enroll their own children in similar activities. As one mother explained:

I enrolled my son [5 years old] in a karate class. He also is taking swimming lessons at the YMCA. My next-door neighbor and me take turns taking the kids to swim lessons. It’s really helpful because sometimes I need to get home later from work and we help each other that way… FGD #6; Mother #27

Likewise, some mothers reported that having close friends with young children motivated them to get together and take their children to parks and playgrounds. For example, one mother described:

Sometimes I am home and a friend will text or call me and say, “Do you want to go to the park with the kids? Having a friend [mother] who also has children about the same age make it easier for everyone …the kids play and we [mothers] talk…It’s just makes it better to have company and you feel like getting out with the kids more… FGD #3; Mother #17

#### Encouraging, praising and providing motivational support

The majority of mothers spoke of offering their child motivational support by encouraging and praising (e.g., “keep going”, “good job”) their children while they are physically active. Mothers felt that this encouraged their children to continue to be physically active. For example:

I think it’s important for parents to be supportive and encourage their children to keep trying, to motivate them to learn and to try and learn new things…humm…to improve. I am a big supporter of my son. He plays soccer and I always watching him play. Cheering, saying good job, do it again…that’s great! He loves when I say, “you played so well”! You are the best. Number 1 &[laughs]! FGD #4; Mother #19

Moreover, several mothers mentioned encouraging their young children’s participation in organized sports. As one mother described:

My son [8 years old] always loved playing soccer, since he was little he would have a soccer ball with him all the time …we enrolled him in a little soccer league and he plays all year around. Last year his team won the town tournament and he got a trophy for the player who scored the most goals … we were so proud of him! We now have a shelf in his room that we say are for his soccer trophies …we want him to continue to play soccer…who knows what’s in his future [laughs]… FGD #3; Mother #13

Furthermore, more than half of the mothers spoke of being proud of their children when they participated in organized sports or physical activities, such as soccer games, swimming, dance classes, etc. They felt that their pride furthered their children’s motivation to be physically active, as one mother explained:

My daughter took swimming lessons at the YMCA and her teacher told me the YMCA had a swim team and that I should consider enrolling her [daughter] … this is her second year swimming with the team and she loves it! My husband and I take her to all the meets and we cheer for her … she feels so proud when she wins and we are even more proud than she is …I feel that us [parents] showing them how proud we are and motivating them to keep doing it is really important, especially when they are young. Now, she wants to swim [team] even during the summer … FGD# 3; Mother #15

#### Watching, supervising, and teaching children how to engage in physical activity

More than half of the mothers spoke of watching and supervising their children engage in PA and that they felt this promoted their children to be active. Several mothers reported supervising their children playing at local parks and playgrounds as the following quote illustrates.

I take my kids to the park and playground when the weather is nice. My little one is learning to ride her bike without the wheels and it’s safer for her to ride her bike in the park. There is a safe paved area in the park and I can walk by her side and help her… FGD #2; Mother #11

Some mothers also spoke of teaching their kids different types of PA such as such as swinging, climbing a tree, riding a bicycle, etc. One mother described:

One day we were at this nice park near a friend’s house and there were a lot of trees…my son was trying to climb a tree, but couldn’t really do it. His friends were used to climbing that tree … he was so upset. So, I took my shoes off and showed him how to climb the tree (laughs)… back in Brazil we grew up climbing trees and it was so nice to see how happy he was after he was able to climb the tree… he kept climbing up and down… he was so proud of himself! FGD #7; Mother #35

In addition, some mothers expressed joy and feeling gratified at being able to help their children learn new activities, and that this inspired their continued guidance in encouraging their children to participate in these activities and offered children new opportunities in PA. As one mother described:

I grew up climbing trees and one day we were at the playground near our house and my daughter was trying to climb a small tree, but couldn’t do it… she was really upset because all the other kids could do… so I helped her and she kept trying and now every time we go to that playground she runs to the tree to show me that she knows how to do it by herself. It makes me so happy to see how proud she’s of herself… we grew up climbing trees [Brazil]… FGD #6; Mother #27

#### Monitoring and setting limits to setting child’s sedentary time

Several mothers spoke of monitoring and setting limits to their children’s screen time (e.g., TV watching or using tablets, iPads, and video game consoles), which mothers perceived as positively influencing their children not to be physically inactive. One mother reported:

I think parents need to be vigilant and make sure the kids are not on their electronics too much. At our house, I tell my kids you can watch TV or play in your iPad for one hour, that’s it. When one hour is up, they need to find something else to do. It’s not easy but as parents we have a responsibility to teach them [children]… FGD #2; Mother #9

#### Prompting child to be physically active

Some mothers mentioned prompting their preschool-age children to be physically active, especially when they thought their children had too much screen time. This is illustrated in the following quotes:

When I see the kids have been inside the house for too long, watching cartoons on their iPads, I tell them to go outside and play. If you let them, they will stay on their iPads all day. So, I tell them “Come on, let’s go outside. Let’s go for a walk…” FGD #1; Mother #4

Sometimes the kids are inside the house and not doing much, so I see the weather is nice outside and I tell them, “Ok, go outside! Go play outside, the weather is nice!” It’s not good to be inside the house the whole day…so, go play outside! FGD #5; Mother #28

### Domain 2: Parenting practices that discourage or inhibit children’s physical activity

Analyses identified four parenting practices within this domain (see Table 2). These parenting practices were: 1) modeling of sedentary behaviors; 2) having rules and restrictions due to safety- and weather-related concerns; 3) limiting child’s outdoor time due to parental time constraints; and 4) restricting child’s outdoor and play time as punishment. These parenting practices are presented below with selected illustrative quotes.

#### Modeling sedentary behavior

As mentioned earlier, the vast majority of mothers spoke of the importance of parents’ being role models for PA, and almost all mentioned parents’ modeling of sedentary behaviors (e.g., parents who do not exercise, parents who have too much screen time) as negatively influencing children’s PA and increasing their sedentary time and contributing to the development of sedentary habits. One mother described:

Children watch and copy what their parents and other adults do. The good habits, and the bad ones too & So, if we as parents are always online, the kids see that and do the same. The other day, I told my daughter, you need to put your iPad away. Do you know what she told me? It’s not fair. If I have to put my iPad away, then you need to put your phone away. She’s only 4-years old. They know; they watch what we do. We need to set a good example… FGD #7; Mother #36

#### Having rules and restrictions due to safety- and weather-related concerns

Many mothers reported having rules and restrictions such as not allowing children play outside without an adult due to safety (e.g., neighborhood safety) or traffic (e.g., busy street) concerns. Mothers acknowledged that these rules and restrictions reduced their children’s PA. These are illustrated in the following comments:

We live in a busy street, so I don’t let the kids play outside or ride their bikes on the sidewalk because I don’t think it’s safe. People drive their cars really fast and I worry about the kids being hit by a car…I don’t think it’s safe. FGD #7; Mother #33

Our street has a lot of traffic and it’s hard to let the kids play outside…my son likes to ride his bike on the sidewalk, but I get nervous because the streets are narrow and cars drive really fast…so I only let him ride his bike in the park. FGD #3; Mother #13

Furthermore, more than half of the mothers stated that weather concerns (cold weather) sometimes made them limit their children’s time outdoors, which they recognized may negatively impact their children’s PA. Mothers also reported their children spend more time outdoors and were more active during the warmer months. One mother explained:

In the winter it’s hard to get the kids outside to play…it gets cold and dark early… so the kids spend more time indoors. My kids are more active during the summer, when the weather is nice and after so many months of winter, we all want to be outside… FGD #4; Mother #22

Some mothers did acknowledge that their children would probably like to play outside during the cold months, but that they themselves did not like being outside during the cold weather, as they were not used to it. One mother explained:

My kids don’t really mind the cold weather. They like playing outside in the snow, sledding & They are growing up with the cold weather and used with the cold [weather]…but, I cannot get used to it. I don’t like the winter and prefer not being outside when it’s cold. I grew up with warm weather all year around…I just can’t get used to the cold [weather]… FGD #4; Mother #18

#### Limiting children’s time outdoors due to parents’ limited time

About half of the mothers reported that their children engaged in sedentary activities indoors (e.g., coloring, playing with toys, watching TV, playing games on tablets, etc.) due to their time constraints because of work obligations or household chores. Some mothers spoke of getting home after a long day at work and still having to do household chores (e.g., cooking dinner, laundry, etc.) and said that it is often easier for them to keep the children indoors instead of taking them outside to play so they can get things done around the house. Others explained that they were tired when they come home from work, and do not always have the energy to take their children out to a park, playground, or outdoors to play. For example, one mother explained:

Sometimes I don’t have time to go out with the kids. By the time I get home from work, need to prepare dinner, do some household chores … it gets dark and late for the kids to go outside and play. So, the kids stay in the house playing & FGD #2; Mother #8

#### Restricting child’s outdoor and play time as punishment

A couple of mothers spoke of making children stay inside, giving time outs (e.g., making child sit to “calm down”), and not allowing children to play outdoors as punishment for what they perceived as bad behavior. This practice was classified as inhibiting young children’s PA.

Sometimes a parent needs to make a child sit down and take a break to calm down and I think that could be a way to keep a child from playing and being active. Like my son, sometimes he just gets too hyper and out of control, so I tell him: Please sit down, just quiet down for a few minutes then you can play again. He just needs a break sometimes… FGD #6; Mother #28

### Domain 3: Social contextual factors influencing parenting practices

Although not the focus of the FGDs, analyses determined that social contextual factors (e.g., income, housing, neighborhood safety, etc.) influenced mothers’ PA parenting practices, and in turn, children’s PA. Some mothers spoke about not being able to provide child with opportunities for PA and active play due to financial constraints and limited space. Furthermore, some mothers mentioned that limited resources kept them from enrolling their children in organized sports activities. One mother said:

I’d like to enroll the little ones [children] in some classes, but sometimes it’s expensive and the classes time are not good for my schedule … when the kids are older it’s easier you can drop them off for soccer and pick them up, but the little ones, you need to stay with them… FGD #3; Mother #16

Moreover, as mentioned earlier, a number of mothers reported having limited space (e.g., apartment building, lack of a backyard) where their children could play safely (traffic and neighborhood safety concerns) and that this inhibited their children’s PA. One mother stated:

We live in an apartment building, so we don’t have a place for the kids to go out and play outside…unless my husband or me are free and can take them out, the kids pretty much stay inside… FGD #4; Mother #20

## Discussion

This study contributes to the scant literature examining PA parenting practices of Brazilian immigrant mothers, a rapidly increasing Latino immigrant subgroup in the US [39]. Analyses identified a number of parenting practices that may encourage or facilitate children’s PA that occurred at the interpersonal-level (e.g., parental modeling of PA, parental engagement and involvement, provision of logistic support; encouragement and praise, etc.) and that appeared to be influenced by social contextual factors. Although parenting practices that may discourage or inhibit children’s PA were identified at the interpersonal level (e.g., parental modeling of sedentary behaviors, discipline, etc.), these practices appeared to be influenced by both environmental (limited space, neighborhood safety, etc.) and social contextual (e.g., work obligations; limited financial resources) factors that affect the day-to-day lives of Brazilian immigrant families. For example, mothers reported implementing rules and restrictions due to safety and weather-related concerns, lacking resources to enroll their children in sports or PA classes, etc. These findings suggest that culturally appropriate parenting- and family-based interventions designed to increase preschool-aged children’s PA should consider the social context of Brazilian immigrant families’ daily life. For example, these interventions could use a family-tailored approach that helps parents develop parenting skills to facilitate and encourage PA for their preschool-aged children. Moreover, developed interventions could provide tailored content to help parents overcome specific contextual barriers experienced by Brazilian immigrant families (e.g., lack of time due to work obligations, safety concerns, limited space) [40,41].

Parental modeling was the most commonly cited parenting practice, and was viewed by mothers in this study as both encouraging (modeling of PA) and discouraging (modeling of sedentary behaviors) children’s PA. Mothers explained that children often mimic their parents’ behavior, for the better (parent being physically active), and for the worse (parents display of sedentary behaviors). These findings concur with previous research suggesting the positive and negative influences of parental modeling on children’s PA and sedentary behaviors [10,22,38,42–44].

Furthermore, the majority of mothers in this study reported that parental engagement and involvement in children’s PA encouraged children to be more physically active, which concurs with previous research conducted among other racial/ethnic groups in the US [45,46]. These findings, combined with mothers’ awareness of both positive and negative influences of parental modeling on children’s PA, are noteworthy and can be used to inform intervention messages.

Additionally, mothers identified providing logistic support, offering verbal encouragement, praise and motivational support, supervising, watching and teaching the child to engage in various types of PA, and prompting child to be physically active as parenting practices that encourage or facilitate preschool-age children’s PA. These findings are consistent with prior studies demonstrating the positive influence of these parenting practices on children’s PA [3,17,22,23,38,47]. Evidence from research with Hispanic parents shows that logistic support can positively impact children’s PA [48–51]. Similarly, previous studies conducted with Hispanic parents have determined that positive reinforcement as measured by a parent’s praising of the child being physically active is positively associated with children’s PA [13,22]. Likewise, parental verbal encouragement of PA positively influences child PA [45,51]. Moreover, studies conducted with parents in Canada, US, and Hong Kong have shown that parents’ believe that supervising and teaching children how to engage in PA is an important strategy that positively influences children’s PA [16,22,38].

Mothers participating in this study also spoke of monitoring and limiting child’s screen time as a strategy to decrease children’s sedentary time and promote children’s PA. Current evidence on the associations between parenting practices and child screen viewing is mixed, with some studies showing a positive influence of this practice on children’s PA, while others show a negative association [52–57]. Current research suggests that the association between parenting practice of setting limits and child’s screen time varies depending on the type of screen time (e.g., TV viewing, computer use, playing video games, using smartphones, etc.) and the frequency of limit setting [53,58]. These findings suggest that interventions designed to promote PA among children in Brazilian immigrant families should also focus on helping parents develop skills needed to limit screen time.

Although mothers participating in this study reported a number of parenting practices that may encourage or facilitate their children’s PA, they also spoke of parenting practices that have been shown in prior research to discourage or inhibit their young children’s PA [47,59–64]. As mentioned previously, mothers felt that that parental modeling of sedentary behavior (e.g., screen use, TV watching, etc.) may inhibit PA. Additionally, mothers in this study reported having rules and restrictions due to safety- and weather-related concerns that limited their children’s outdoor time and active play. This finding is similar to that of prior research conducted with other Latino ethnic groups [59,60,63,64]. Furthermore, consistent with prior research a lack of time and lack of safe space (e.g., backyard) for child to play outdoors and lack of resources were identified as limiting children’s PA [10,22,47,65,66]. These findings are important and suggest modifiable PA parenting practices that can be addressed in culturally sensitive interventions designed to promote PA in preschool-age children of Brazilian immigrant families living in the US. Although more research is needed, these findings suggest potential targets for interventions designed to promote PA among preschool-aged children of Brazilian immigrant families living in the US. Nonetheless, additional qualitative research with Brazilian immigrant fathers and Brazilian parents from other communities across the US should be considered. Although the study sample included approximately 51% of participants with household income under US$ 40,000, parents of lower socioeconomic status should also be considered. Furthermore, future research should also consider employing objective measures to assess PA parenting practices Brazilian immigrant parents use to promote PA. These studies could employ cross-sectional or longitudinal study designs to assess the associations between PA parenting practices and preschool-age children’s PA.

## Limitations

Study findings should be considered in light of some limitations. Findings are based on a non-random, purposeful and a relatively small sample of low-income Brazilian-born immigrant mothers in two cities in MA, which limits generalizability. Participating mothers might have had a heightened interest and awareness regarding the focus group topics. Moreover, the use of snowball sampling to recruit participants might have resulted in the recruitment of study participants who share similar beliefs and practices related to PA. Thus, further research is needed to increase generalizability and to explore whether results apply to a broader group of Brazilian immigrants. Finally, the present study included only mothers, and this is a limitation given the increasing evidence suggesting the importance of including both parents in child health promotion and obesity prevention research and interventions [10,24,38,42,55]. Future research can address these limitations by exploring PA parenting practices used by Brazilian mothers and fathers from other communities across the USA, selecting a larger sample size and employing multiple data-collection methods, including both qualitative and quantitative methods.

## Conclusions

The present study provides new information on PA parenting practices of Brazilian-born immigrant mothers of preschool-aged children living in the US that encourage and/or discourage children’s PA. Although additional research is needed, study’s findings may provide important targets to consider when designing interventions to promote PA among preschool-aged children of Brazilian immigrant families and can inform specific intervention components and strategies for this particular ethnic group. Future research should further explore PA parenting practices of Brazilian immigrant parents including quantifying PA parenting practices that encourage and discourage PA, as well as examining the influence of fathers’ PA parenting practices on young children’s PA. This information is needed to identify factors amenable to interventions and to design culturally appropriate parenting and family-based interventions targeting the home environment of Brazilian children of immigrant families and designed to meet the specific needs of this ethnic group.

## Supporting information

S1 Table Socioeconomic and demographic characteristics of the sample. SED-Sample_PONE.docx.
(DOCX)
