# Supplementary material for: Parenting practices that may encourage and discourage physical activity in preschool-age children of Brazilian immigrant families: A qualitative study
Source: PLoS One. 2019 Mar 20;14(3):e0214143. doi: 10.1371/journal.pone.0214143 (PMC6426301; doi:10.1371/journal.pone.0214143)
Supplement: S1 Table — SED-Sample_PONE.docx. (DOCX) [file pone.0214143.s001.docx]

**Table 1. Socio-demographic and acculturation characteristics of focus group participants (n = 37)**

**Age Mean +SD (range) N (%)**

35.3 + 2.8 (26 – 41) 37 (100)

**Race (Ethnicity)**

Latino (Brazilian) 37 (100)

**________________________________________________________________________________________________________**

**Born in Brazil**

Yes 37 (100)

No 0

**Regions and States of Brazil of Origin**

**Southeast**

Espirito Santo 10 (27)

Minas Gerais 22 (59.5)

Sao Paulo 1 (2.7)

**South**

Santa Catarina 2 (5.4)

**Midwest**

Goias 1 (2.7)

Mato Grosso 1 (2.7)

**Years in the United States Mean + SD**

6.7 + 2.84 37 (100)

**Primary language spoken at home**

Portuguese 37 (100)

**Marin scale acculturation score**  **Mean + SD**

1.43 + 0.77 37 (100)

**Marital status**

Single 1 (2.7)

Married 34 (92)

Divorced/Separated 2 (5.4)

**Number of children in household Mean + SD (range)**

2.2 + 1.4 (1–4 children)

**Education level**

Less than high school 10 (27)

High school degree 21 (56.8)

GED^*^  1 (2.7)

College 4 (10.8)

Missing 1 (2.7)

**Household annual income**

> $40K/year < $60K/year 18 (49)

< $40K/year 19 (51)

^*^General Education Diploma
